# DNA Damage, Repair, and Cancer Metabolism

**DOI:** 10.3389/fonc.2018.00015

**Published:** 2018-02-05

**Authors:** Marc-Olivier Turgeon, Nicholas J. S. Perry, George Poulogiannis

**Affiliations:** ^1^Department of Cancer Biology, Institute of Cancer Research, London, United Kingdom; ^2^Division of Computational and Systems Medicine, Department of Surgery and Cancer, Imperial College London, London, United Kingdom

**Keywords:** metabolism, DNA repair, DNA damage, cancer, reactive oxygen species

## Abstract

Although there has been a renewed interest in the field of cancer metabolism in the last decade, the link between metabolism and DNA damage/DNA repair in cancer has yet to be appreciably explored. In this review, we examine the evidence connecting DNA damage and repair mechanisms with cell metabolism through three principal links. (1) Regulation of methyl- and acetyl-group donors through different metabolic pathways can impact DNA folding and remodeling, an essential part of accurate double strand break repair. (2) Glutamine, aspartate, and other nutrients are essential for *de novo* nucleotide synthesis, which dictates the availability of the nucleotide pool, and thereby influences DNA repair and replication. (3) Reactive oxygen species, which can increase oxidative DNA damage and hence the load of the DNA-repair machinery, are regulated through different metabolic pathways. Interestingly, while metabolism affects DNA repair, DNA damage can also induce metabolic rewiring. Activation of the DNA damage response (DDR) triggers an increase in nucleotide synthesis and anabolic glucose metabolism, while also reducing glutamine anaplerosis. Furthermore, mutations in genes involved in the DDR and DNA repair also lead to metabolic rewiring. Links between cancer metabolism and DNA damage/DNA repair are increasingly apparent, yielding opportunities to investigate the mechanistic basis behind potential metabolic vulnerabilities of a substantial fraction of tumors.

## Introduction

Over the past decade, a renewed interest in cancer metabolism has emerged. The idea originated from the work of Otto Warburg and colleagues—first published in the 1920s—who noticed the propensity for cancer cells to consume increased quantities of glucose (1, 2). We now understand that there is extensive metabolic rewiring in cancer cells, and we are starting to decipher how cancer cells reprogram their metabolism to adapt to changes in their microenvironment and support their high metabolic needs (3–5). Cancer metabolism is a broad topic and our current understanding of how metabolic reprogramming is linked to malignant transformation has been extensively reviewed elsewhere (6–8). Therefore, in this review, we decided to focus on the connections between cell metabolism and another major aspect of cancer biology, DNA-repair/DNA-damage pathways.

The major hallmarks, as described by Pavlova and Thompson (7), include: deregulated glucose and amino acid uptake, opportunistic ways of acquiring nutrients, use of metabolic intermediates for biomass and nicotinamide adenine dinucleotide phosphate (NADPH) synthesis, increased demand for nitrogen, and alterations in metabolite-driven gene expression and interaction with the microenvironment (7, 9). Importantly, the wide differences in metabolic dependencies across cancer types, or stage of progression, as well as the dynamic shifts between metabolic pathways, make the study of cancer metabolism and the development of new therapies targeting these pathways very challenging.

**Figure 1:**
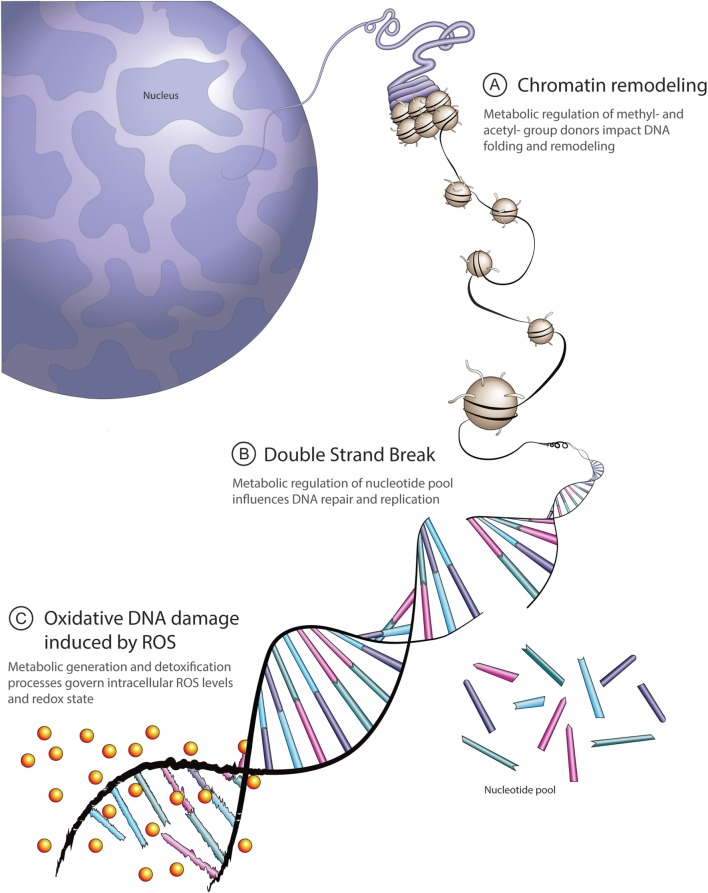
Overview of principal links between cell metabolism and DNA repair. **(A)** Methyl-group donors from the S-adenosylmethionine pathway and acetyl-donors from citrate cycle-derived acetyl coenzyme A contribute to dynamic chromatin packaging and remodeling essential to DNA double-strand repair. **(B)** Metabolic intermediates derived from glucose, glutamine, and aspartate are required for *de novo* nucleotide synthesis. The ready availability of a pool of nucleotides facilitates appropriate DNA repair and replication. **(C)** Intracellular reactive oxygen species (ROS) levels reflect a balance between generation and detoxification. A principal ROS detoxification mechanism involves reduced glutathione (GSH), determined by glutamine and cysteine availability, as well as NADPH levels. High ROS-induced DNA damage leads to excessive burden on the DNA repair machinery.

In addition to the accepted role for cell metabolism in cancer, it is well established that DNA-repair/DNA-damage pathways are important in cancer progression because dysregulation leads to higher levels of genomic instability, increased mutation rate, and enhanced intra-tumor heterogeneity (10–13). There are currently three principal mechanisms through which changes in cell metabolic status are thought to have an influence on DNA-damage/DNA-repair pathways: chromatin remodeling, double-strand break (DSB) repair, and redox homeostasis (Figure 1). Uncovering new links between these important aspects of cancer biology might lead to the development of new targeted therapies in DNA-repair deficient cancers or even improving the efficacy of existing therapies, such as PARP inhibitors, anthracyclines, and platinum salts. In this review, we examine work seeking to uncover the links between DNA-repair/DNA-damage and cell metabolism.

### Metabolic Status Affects DNA Folding and Repair Pathways

**Figure 2:**
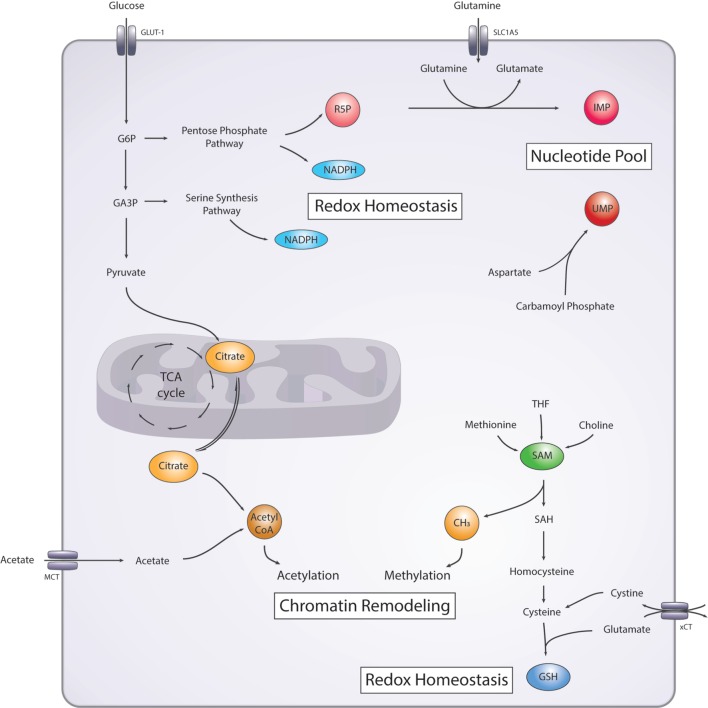
Simplified diagram of the main metabolic pathways involved in DNA damage/repair. **Nucleotide pool:** nucleotide precursor ribose-5-phosphate (R5P) is generated from the pentose-phosphate pathway (PPP). Purines and pyrimidines precursors, inosine monophosphate (IMP), and uridine monophosphate (UMP), respectively are synthesized from glutamine, and aspartate and carbamoyl phosphate, respectively. **Redox homeostasis:** nicotinamide adenine dinucleotide phosphate (NADPH) is generated from the PPP and the serine synthesis pathway. Glutathione (GSH) is generated from cysteine and glutamate. **Chromatin remodeling:** methyl-group donors (CH_3_) are generated from the S-adenosyl methionine (SAM) pathway and acetyl-group donors (acetyl coenzyme A) are generated from the citrate cycle or from acetate. GLUT-1, glucose transporter 1; SCL1A5, alanine, serine, cysteine-preferring transporter 2 (ASCT2); G6P, glucose 6-phosphate; GA3P, glyceraldehyde 3-phosphate; SAH, S-adenosyl homocysteine; xCT, cystine-glutamate antiporter; MCT, monocarboxylate transporter family.

The first link between DNA repair and cell metabolism involves DNA folding and organization. Chromatin packaging and remodeling, through different histone posttranslational modifications—including acetylation, methylation, phosphorylation, and ubiquitination, as well as through DNA modifications such as methylation—can regulate gene expression levels by modulating the access to DNA of different protein complexes (14, 15). Interestingly, similar mechanisms can also regulate access of DNA-repair proteins to the DNA double-helix (16). When repairing DSBs, the first step involves unfolding DNA to allow access of the repair complexes to the DSB (17). There is a growing body of evidence suggesting that these mechanisms can in fact regulate the choice of DNA-repair pathway (i.e., homologous recombination), or non-homologous end-joining used to repair the DSB (16). Substrates added to histones or DNA are derived from metabolic intermediates (18–20) (Figure 2). For example, methyl-group donors mostly come from the S-adenosylmethionine (SAM) pathway (18), while the sole acetyl-group donor is acetyl coenzyme A (acetyl-CoA), known for the transfer of its acetyl group to oxaloacetate to form citrate and start the tricarboxylic acid (TCA) cycle (21). It has been shown that expression levels of the enzyme, ATP citrate lyase, can regulate the availability of acetyl-CoA in the cell (22). Restricting the amount of acetyl-group donors can disrupt proper DNA organization and have an impact on DNA folding and DNA remodeling essential to successful DNA DSB repair (22, 23). Acetyl-CoA can also be generated from acetate and CoA from the acetyl-CoA synthetase 2 enzyme. In fact, under metabolic stress conditions in which oxygen and lipids are deprived, acetate-generated acetyl-CoA is enhanced and acetate-derived carbons are incorporated in lipid synthesis (24, 25). Acetate-derived acetyl-CoA has also been shown to be involved in histone acetylation, suggesting that acetate availability could influence histone acetylation in cancer cells (26).

Additionally, changes in the activity of the SAM pathway due to availability of necessary nutrients or substrates also influence DNA or histone methylation by regulating the pool of methyl-donors (27). The SAM pathway is interconnected with methionine, tetrahydrofolate (THF), one-carbon metabolism, and choline pathways. These pathways tend to compensate for one another; however, depletion of choline, folate, or methionine can still have an impact on the final SAM concentration (18). Such changes in the SAM pathway not only influence gene expression of cancer-associated genes through epigenetic modifications but can also impact DNA folding during DNA repair processes. The latter can also be influenced directly by metabolic intermediates such as fumarate. DNA-PK dependent activation of fumarase contributes to enhanced local generation of fumarate, which in turn promotes DNA repair *via* inhibition of KDM2B-mediated histone demethylase activity (28). Accumulation of fumarate can also lead to epithelial-to-mesenchymal transition and poor clinical outcome (29). Since modifications in histone and DNA are important for proper repair of DNA DSBs (30), changes in nutrient and substrate levels that disrupt these modifications are bound to affect DNA-repair pathways.

### Nucleotide Levels Affect DNA-Repair Potential of Cancer Cells

Another major mechanism upon which cell metabolism can regulate DNA-repair/DNA-damage is through the regulation of the pool of nucleotides used for DNA replication and repair (31, 32). Many different metabolic pathways are involved in *de novo* nucleotide synthesis and can have an impact on the levels of intracellular nucleotides available (Figure 2) (31, 33, 34). An important precursor in the synthesis of the ribose backbone, essential for both purines and pyrimidines, comes from ribose-5-phosphate, an intermediate of the pentose-phosphate pathway (PPP) (31). Briefly, the PPP utilizes glucose-6-phosphate (G6P), an intermediate of glycolysis, which can be redirected to generate metabolic intermediates necessary for nucleotide and protein synthesis, as well as generation of NADPH. The PPP is a major focus in cancer metabolism, and it has been carefully reviewed elsewhere (31, 35, 36). The increased glucose consumption observed in cancer cells has been shown, at least in some cancers, to be used to fuel the PPP, where it can potentially be used to generate reducing power in the form of NADPH and nucleotide precursors (32). Additionally, other metabolic pathways are involved more specifically in purine or pyrimidine ring synthesis. The amide group of glutamine is essential in two steps of inosine monophosphate synthesis, an intermediate in *de novo* purine synthesis, and in one step of uridine monophosphate synthesis, an intermediate in *de novo* pyrimidine synthesis (37, 38). In glioblastoma, it has been shown that α-ketoglutarate is diverted out of the TCA cycle to synthesize more glutamine through the glutamine synthetase enzyme. The resulting glutamine is then used for *de novo* purine synthesis (39). Furthermore, aspartate is essential for the synthesis of pyrimidines, and glycine is also essential for purine synthesis (38). Loss-of-function mutations in arginosuccinate synthase 1, which leads to reduced arginine production in the urea cycle, promotes the use of aspartate for pyrimidine synthesis (33). Aspartate is mostly synthesized from glutamate and oxaloacetate. Therefore, the availability of glutamine and/or other amino acids/metabolic substrates can influence the amount and ratio of nucleotides produced in a cell and might provide a regulatory mechanism for DNA-repair.

### Deregulated Redox Homeostasis Promotes DNA Damage

The metabolic regulation of reactive oxygen species (ROS) levels is the third and final link that we address here between DNA-repair/DNA-damage and cell metabolism. High ROS levels affect many aspects of tumor biology and, here, we focus on their role in inducing DNA damage and genomic instability. Most of the DNA lesions that are formed by ROS-induced damage are single strand breaks (SSBs) that can be repaired through nucleotide or base excision repair (NER/BER) (40). However, these SSBs can lead to stalling of the replication fork or error in replication, ultimately leading to DSBs (40). The accumulation of oxidative DNA damage increases the burden on the DNA-repair machinery. Tight regulation of cellular redox stress is essential, since high ROS levels can lead to oxidative stress and oxidative damage of proteins, DNA, and lipids, while a certain level of ROS is essential for activating signaling pathways involved in multiple biological processes (41–43). Cells have evolved a number of ways to balance ROS levels (Figure 2). Glutathione (GSH) is one of the major ROS-scavenging molecules (44), and it occurs in two versions; the reduced form, sulfhydryl GSH, and the oxidized, glutathione disulfide (GSSG). The enzyme GSH peroxidase catalyzes the reduction of H_2_O_2_ to water and lipid hydroperoxides to their corresponding lipid alcohols and in turn oxidizes GSH to GSSG. The reverse reaction is catalyzed by the enzyme glutathione reductase, also known as glutathione-disulfide reductase using the reducing potential of NADPH (45).

Glutathione is synthesized in the cytoplasm from cysteine and glutamate, and cysteine availability is the rate-limiting step of the synthesis reaction (46, 47). Cysteine can be synthesized from serine through the transsulfuration pathway (47), or it can be transported into the cells as cystine in exchange for glutamate using the cystine-glutamate antiporter xCT (39). Interestingly, mTORC2 can phosphorylate and inhibit the xCT antiporter, therefore providing a mechanism to regulate intracellular cysteine levels (48). Furthermore, it has been shown that, in some cancer types, growing cells in DMEM containing cystine leads to xCT dependent glutamine dependency *in vitro*, providing evidence for context dependent changes in metabolism (5). The main source of NADPH comes from the PPP and a significant amount also comes from the serine synthesis pathway through the THF pathway. Both harness the energy from the catabolism of metabolic intermediates to generate NADPH (31, 47). Interestingly, an important regulator of ROS levels is the transcription factor nuclear factor E2-related factor 2 (NRF2) that has been shown to directly regulate the key serine synthesis enzymes (PHGDH, PSAT1, ATF4) (49). This provides a potential mechanism through which NRF2 can regulate ROS levels by influencing NADPH and GSH levels. Importantly, the DNA-repair protein, BRCA1, regulates NRF2, providing an additional link between DNA repair pathways and ROS levels (50). Overall, there are still many open questions with regards to how cancer cells can rewire their metabolism based on different nutrient availability, but there is growing evidence to suggest that maintaining cellular redox homeostasis plays a major role on DNA-damage/DNA-repair pathways.

### DNA Damage Response (DDR) Triggers Metabolic Rewiring

While DNA-repair pathways can be influenced by cellular metabolic status and nutrient availability in the tumor microenvironment, accumulation of DNA damage due to extrinsic and intrinsic genotoxic stress, or deficient DNA repair can also cause abrupt rewiring of cell metabolism. Cells have evolved the DDR pathway (DDR) to monitor this genotoxic stress and maintain accurate transmission of genetic information to subsequent generations. The DDR can, therefore, halt cell-cycle progression, induce DNA-repair mechanisms, or trigger programmed cell death when DNA damage is irreparable (51). Ataxia telangiectasia mutated (ATM) and ataxia telangiectasia and Rad3-related (ATR) kinases are two key enzymes in the recognition of DNA damage and implementation of the DDR (51, 52). Upon activation by DNA-damage, ATM and ATR generate a second wave of phosphorylation that impacts many downstream effector proteins. An important aspect of DDR, driven by ATM and ATR activation, is the induction of metabolic rewiring to promote the resolution of genotoxic stress. ATM has been shown to activate the PPP through induction of the rate-limiting enzyme glucose-6-phosphate dehydrogenase (G6PD), to support the synthesis of reducing power in the form of NADPH, and generate ribose-5-phosphate for nucleotide synthesis (53, 54).

Increased carbon flux to the PPP from glucose derivatives is driven not only by increased glucose consumption often observed in cancer cells (55, 56) but also through ROS-mediated inactivation of many glycolytic enzymes, notably PKM2 that promotes flux into the oxidative arm of the PPP as well as serine biosynthesis (57). Many studies have shown increased glucose dependency following DDR (58), and these data demonstrate the challenge posed to cancer cells under genotoxic stress. They need to contain ROS levels, enhance nucleotide synthesis to repair DNA damage, and inhibit cell-cycle progression until resolution. To this end, SIRT4 activation upon DDR has been shown to inhibit glutamine consumption in HepG2, HeLa, HEK293T cells, and in lung tissue (58). The reduced glutamine consumption or, more specifically, reduced anaplerosis of glutamine into the TCA cycle, confers improved cell survival compared to SIRT4 knockout cells that cannot activate this response (58). While this study suggests that DDR undoubtedly causes changes in metabolism, conflict in the literature indicates that the nature of these changes is context- and cell type-dependent. For example, the concept that cells undergoing genotoxic stress need to synthesize more nucleotides to repair the DNA damage contradicts the reduced glutamine consumption. As mentioned previously, the amide group of glutamine is essential for purine and pyrimidine *de novo* synthesis. Other interesting points to note are that in non-cancerous tissues/cells, there are increases in fatty acid oxidation (FAO) and oxidative phosphorylation (OXPHOS) in response to acute or chronic genotoxic stress (59). Increased FAO/OXPHOS is driven by AMP kinase (AMPK) activation resulting from the depletion of ATP. In the context of that study, cells are depleted of ATP because of the utilization of NAD+ by the DNA repair enzyme PARP-1. PARP-1 generates PAR chains from NAD+ and PARylates DNA to induce repair. This leads to depletion of NAD+, reduction of ATP synthesis, and activation of AMPK (59). This study further supports that DNA damage impacts cell metabolism. However, given the current literature, it is still unclear how a cell’s nutrient availability can dictate the nature of its metabolic rewiring.

### Metabolic Changes Driven by DDR Gene Mutations

The p53 protein encoded by the *TP53* gene is another key player in DDR through its role in DNA repair and cell-cycle regulation (60–62). While p53 levels are kept low under normal conditions, upon DNA damage or other cellular stress, p53 is activated by phosphorylation events that prevent its degradation (60). Wild-type p53 activation regulates cell-cycle entry by transcriptionally repressing cyclin B and CDC25B, and it also plays an important role in DNA-repair. More specifically, it determines whether or not to repair the DNA or trigger senescence or apoptosis. p53 also plays many modulatory roles in the metabolic rewiring of cells. Among these, is its regulation of glycolysis through transcriptional activation of the *TP53*-induced glycolysis and apoptosis regulator (TIGAR) protein (63). TIGAR inhibits glycolysis by dephosphorylating fructose-2,6-biphosphate (F2,6B) leading to degradation of F2,6P and inhibition of phosphofructo kinase 1, an enzyme integral to glycolysis. p53 has also been shown to inhibit other enzymes of glycolysis as well as directly repress the expression of glucose transporters, GLUT1 and GLUT4 (61, 64). One might surmise that this impediment in glycolysis potentially leads to a surplus in glycolytic intermediates, leading to redirection toward the PPP. However, p53 has also been shown to inhibit the flux of glucose to the PPP by directly binding to G6PD and preventing its active dimer formation (61).

In addition, p53 has been shown to transcriptionally regulate proteins involved in the electron transport chain and mitochondrial stability, therefore, providing a mechanism to regulate OXPHOS (61, 65). Together, these data suggest that the role of p53 in metabolic regulation is context and tissue dependent. Importantly, *TP53* is one of the most mutated genes in cancer and many gain-of-function mutations have been described, leading to different changes in p53 depending on the mutation and the context. For example, mutant p53^3KR^ cannot induce cell-cycle-arrest, senescence, or apoptosis, but it can still regulate metabolic target genes resulting in decreased ROS levels and reduced glycolytic flux (61, 66). Therefore, mutations in DDR or DNA-repair genes may result from the application of selective pressures established by the metabolic niches of cancer cells.

Other genes involved in DNA-repair are also commonly mutated in cancer (13). Some of the most well-known DNA-repair genes associated with cancer are *BRCA1* and *BRCA2* but mutations in other genes such as *ATM, ATR*, and *CDK12* [for a more extensive list, see Ref. (13)] also lead to DNA-repair deficient cancers (13, 67–70). Some cancers with a similar phenotype to DNA-repair mutant cancer do not harbor any mutations in those pathways. Together, these DNA-repair deficient cancers have been described as having a “BRCAness” phenotype (13, 71). Some metabolic changes have been described in the context of *BRCA*1 or *BRCA2* mutations (50, 72–74); however, it is still unclear whether these are due to their DNA-repair function or some alternative role. Since connections have been drawn between metabolism and DNA-repair, it seems likely that the “BRCAness” phenotype and *BRCA1/2* mutations would be associated with changes in metabolism. Again, as with most other mutations found in cancer, the metabolic changes associated with these DNA repair defects might reflect the context and tissue of origin.

## Conclusion

Historically, cancer has been thought of as a genetic disease driven by the accumulation of multiple mutation “hits” (75–77). However, in recent years, this paradigm has begun to shift, and cancer is often regarded as a “metabolic disease” (78, 79), which is influenced by complex interactions between the tumor and its microenvironment. Therefore, when examining data suggesting that mutations lead to the rewiring of cancer metabolism, in fact, it may be more pertinent to question whether these mutations arise in the first place from selective pressures applied by extrinsic metabolic factors in the tumor microenvironment. In a noteworthy example, while KRAS-mutant cancers exhibit resistance to low-glucose growth conditions, glucose deprivation has been shown to drive the accumulation of novel KRAS mutations in KRAS wild-type cancers (80). This indicates that extrinsic factors, such as the availability of essential nutrients, directly influence the fate of resulting genetic alterations that confer growth and survival advantages of cancer cells in their given microenvironment.

With regards to mutations in DNA-repair genes, it is possible that the highly dynamic and fluctuating conditions encountered in the tumor microenvironment provide an evolutionary pressure to select for cancer cells that have heightened genomic instability and are more proficient at adapting to these changes as a result. Consistently, it has been shown that tumors with increased heterogeneity are less responsive to therapy and behave more aggressively than tumors arising from single clonal populations (81–83). As we have discussed, a multitude of extrinsic metabolic factors affects DNA repair and, subsequently, genomic stability (Figures 1 and 2). Therefore, when considering future strategies to refine existing or develop novel cancer therapies, it will be essential at all stages of research and drug design to take account of the dynamic interplay between microenvironment and metabolic factors that are proving to influence treatment efficacy so substantially.

## Author Contributions

MOT and GP wrote the manuscript. MOT and NP designed the figure. NP drew the figure and contributed to the revision of the manuscript, and bibliography.

## Conflict of Interest Statement

The authors declare that the research was conducted in the absence of any commercial or financial relationships that could be construed as a potential conflict of interest.
